# Physical Strain During Walking in Adults With Cerebral Palsy: A Construct Validity Study

**DOI:** 10.1016/j.arrct.2026.100617

**Published:** 2026-03-25

**Authors:** Sandra Linnea Klund-Hansen, Terje Gjøvaag, Eivind Lundgaard, Arve Opheim, Linda Rennie

**Affiliations:** a Department of Rehabilitation Science and Health Technology, Faculty of Health Sciences, OsloMet-Oslo Metropolitan University, Oslo, Norway.; b Section for Rehabilitation Technology, Centre for Research and Education, Sunnaas Rehabilitation Hospital, Bjørnemyr, Norway.; c Department of Clinical Neuroscience and Physiology, University of Gothenburg, Gothenburg, Sweden

**Keywords:** 6-minute walk test, Adults, CP (cerebral palsy), Mobility, Oxygen consumption, Quality of life, Rehabilitation, Validity

## Abstract

**Objective:**

To describe the physical strain of walking in adults with spastic cerebral palsy (CP) and investigate the construct (known-groups, convergent, and discriminant) validity of physical strain.

**Design:**

Cross-sectional design.

**Setting:**

Rehabilitation hospital.

**Participants:**

A total of 94 ambulant adults (N=94) (41 men) with spastic CP.

**Interventions:**

Not applicable.

**Main Outcome Measures:**

Peak oxygen uptake (VO_2_peak), physical strain (%VO_2_peak), 6-minute walk test (6MWT), mobility (Timed Up and Go Test), and quality of life (QoL).

**Results:**

The overall physical strain was median (interquartile range) 50% (18), mean (SD) VO_2_peak of 36.2 (8.6), and median (interquartile range) 6MWT distance of 553 m (144). The physical strain test was significantly different between Gross Motor Function Classification System levels I and II (*P*=.002) and between Gross Motor Function Classification System levels I and III (*P*≤.001), and distinguished acceptably between unilateral and bilateral CP subgroups (area under the curve=0.739; *P*<.001). The association between physical strain and 6MWT was high (*r*=−0.716; *P*< .001), the association with timed Up and Go Test was weak-to-moderate (*r*=0.490; *P*<.001), and the association with QoL was very weak and nonsignificant (*r*=0.157; *P*=.129).

**Conclusions:**

Physical strain of walking in adults with spastic CP increased with decreasing gross motor function, and was significantly different between known-groups of adults with spastic CP. This study confirmed the hypotheses of a strong inverse association between physical strain and walking capacity. The weak-to-moderate association with functional mobility and the lack of association with QoL support divergent validity. Our results support the use of physical strain for adults with CP, inversely reflecting functional walking capacity, in clinical practice and research.

Cerebral palsy (CP) covers a group of permanent disorders attributed to nonprogressive disturbances in the developing fetal or infant brain^1^ and is often characterized by motor impairments that limit mobility.^2^^,^^3^ As individuals with CP age, many experience a decline in walking ability,^4^, ^5^, ^6^ earlier and more pronounced than expected.^7^, ^8^, ^9^

To capture how demanding walking is, physical strain has been conceptualized as the oxygen consumption during walking at self-selected walking speed (SSWS) expressed as a proportion of an individual’s peak oxygen consumption (%VO_2_peak).^10^ Research in children and adolescents with CP demonstrates increased physical strain during walking,^10^, ^11^, ^12^ because of both high oxygen consumption (oxygen consumption during self-selected walking speed [VO_2_walk]) and a low maximal aerobic capacity (VO_2_peak).^10^ Emerging evidence suggests similar patterns among young adults with CP.^13^ As this construct combines the oxygen demand of walking with the individual’s VO_2_peak, it offers a more comprehensive indicator of the functional load of walking than oxygen consumption (mL/kg/min) alone.

Previous studies in children^10^ and younger adults with CP^13^ suggest that the physical strain of walking differs between functional levels.^13^ However, more comprehensive validity testing in adults with CP is needed. Therefore, this study aims to describe physical strain during walking in adults with spastic CP and to evaluate its construct validity.^14^ Specifically, we will examine known-group differences, hypothesizing higher strain with higher Gross Motor Function Classification System(GMFCS) levels, and bilateral versus unilateral CP subtypes. Investigating convergent validity with established functional capacity measures, hypothesizing a strong inverse association with 6-minute walk test distance,^15^ as it is shown to be somewhat related to both maximal aerobic capacity^16^ and functional limitations such as hamstrings length^17^ in adults with CP. In addition, discriminant validity with adjacent but distinct constructs, hypothesizing a moderate association with short-duration functional mobility tasks such as the Timed Up and Go (TUG) test, as it includes more strength, coordination, and balance^18^ and is not highly dependent on maximal capacity to the same extent as physical strain. Finally, we hypothesize a weak association with a broader health outcome, quality of life (QoL).

## Methods

### Study design

This cross-sectional study is part of the “Cost of Walking in Adults With Cerebral palsy” study^19^ at Sunnaas Rehabilitation Hospital.

### Ethical approval

Before commencing data collection, data handling approval was ensured by the Norwegian Agency for Shared Services in Education and Research (SIKT no. 655023), and the study was approved by the Regional Committee for Medical and Health Research Ethics in Norway (REK no. 530205).

### Participants

**Fig 1:**
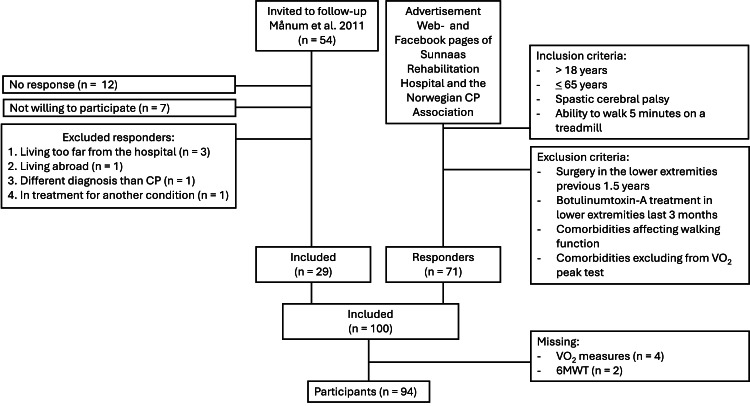
Flow chart of the inclusion process, with inclusion and exclusion criteria. Abbreviations: CP = Cerebral Palsy, and 6MWT = Six-minute walk test.

Participants were recruited through follow-up from a previous study,^20^ advertisements on the Sunnaas rehabilitation hospital and the Norwegian Cerebral Palsy Associations website and Facebook pages, and during hospital admission. See the flowchart (fig 1) for the recruitment process, inclusion, and exclusion criteria.

The study was powered for the convergent validity hypothesis, assuming a 2-sided alpha level of 5%, a 80% power level, and expected correlation coefficients ranging from moderate to large (*r*=0.3-0.5).^21^ Estimates indicate that a total sample size of 85 is needed to detect a moderate correlation, and 29 to detect a high correlation. We therefore aimed to include >85 participants to ensure adequate power for the primary hypothesis.

### Variables

Demographic and clinical characteristics were self-reported, including age, sex, CP type (unilateral or bilateral), physiotherapy usage, exercise frequency, and use of walking aids, and are presented in table 1.

**Table 1:** Demographic and clinical characteristics for all participants.

Characteristic	All participants (n=94)	GMFCS Level I (n=64)	GMFCS Level II (n=22)	GMFCS Level III (n=8)
Sex (M/F), n	41/53	28/36	10/12	3/5
Age, y, median (IQR)	40 (19)	38 (17)	40.5 (23)	47.5 (30)
Body mass index (kg/m^2^), mean (SD)	25.6 (5.8)	25.1 (4.3)	26.2 (4.5)	27.5 (6.8)
CP type, unilateral/bilateral, n	59/35	52/12	7/15	0/8
Physiotherapy,* n				
Never	41	30	10	1
Seldom	26	18	7	1
Often	27	16	5	6
Exercise frequency,* n				
Never	10	6	3	1
Seldom	13	7	5	1
Often	71	51	14	6
Walking aids usage,† n				
None	37	33	4	0
Orthopedic device	46	29	14	3
Walking aids	15	2	5	8
Wheelchair	13	2	5	6

NOTE. Parametric data presented with mean ± SD and nonparametric data distributed presented with median (IQR).

Abbreviation: GMFCS, Gross Motor Function Classification System; IQR, interquartile range.

⁎ Never = never, seldom = seldom or monthly, often = every week or several times a week.

† Orthopedic device = orthotic shoe wear and/or orthosis, walking aids = crutches and/or walker.

The GMFCS^22^ level was self-reported and double-checked with additional information on gross motor function from the participants, and the use of walking aids during the 6-minute walk test (6MWT). The validity and reliability of the GMFCS classification in adults with CP have been established.^22^, ^23^, ^24^

### Treadmill walking tests

The test procedures were performed at the clinical physiological laboratory at a specialized rehabilitation hospital by experienced test personnel on a treadmill (PPS med series).^a^ Because of walking impairments and safety concerns, all participants were permitted to hold onto the handrail of the treadmill with lightweight support if necessary.

Heart rate was measured using a 3-lead ECG monitor (Tango M2).^b^ Throughout the 2 tests, oxygen uptake (VO_2_ mL/min), carbon dioxide output (VCO_2_ mL/min), and minute ventilation (VE; L/min) were measured continuously using a computerized standard open circuit technique breath-by-breath spirometer (Quark CPET).^c^ Blood lactate was measured from capillary blood taken from a fingertip immediately after the VO_2_peak test and after 2-3 minutes (Biosen C-Line).^d^

The spirometer was calibrated before each test, using a 3-L calibration syringe. For calibrating the gas analyzers, medically certified calibration gases (16% O_2_/5% CO_2_) and room air were used. The lactate analyzer was calibrated with a 12.00 mmol/L La- solution before each blood sample.

### SSWS test

The participants’ SSWS was estimated using a modified version of the procedure by Martin et al,^25^ at 0% inclination on a treadmill. The participants started walking at 0.3 m/s. Then, speed was slowly increased until the SSWS was identified and then upheld for 5 minutes. To ensure steady state oxygen uptake, the mean oxygen uptake (VO_2_walk [mL/kg/min]) from the fourth and fifth minute of the SSWS test was extracted, as performed by Darter et al.^26^ To evaluate if the participants chose their preferred SSWS, that is, the most energy-efficient walking speed,^27^ the participants were asked to walk at speeds 20% slower and faster than their SSWS.^26^ Oxygen cost of walking (mL/kg/m) was calculated by dividing VO_2_walk with walking speed (m/s) for all 3 walking speeds.

### Maximal exercise test

A maximum exercise test measuring VO_2_peak (mL/kg/min)^28^ was performed on a treadmill after a 10-minute break, following the Modified Balke Protocol.^29^ Starting at SSWS with 2% incline, and with a gradual increase in incline of 2% per minute, until voluntary exhaustion. If the incline reached 20%, the pace was increased by 10% of the SSWS each minute. Verbal encouragement was given.

To evaluate if the maximum exercise test was met, 2 of the 4 following criteria for maximum effort needed to be met: the age- and sex-related reference values for respiratory exchange ratio and blood lactate (La-),^30^ a VO_2_ plateau (≤2 mL/kg/min rise in VO_2_ despite increased workload at maximal intensity) evaluated by a trained clinical staff member or ≥90% of predicted maximal heart rate (HR max), using the formula (211−[0.64 × age]).^31^^,^^32^ Norwegian reference values for VO_2_peak were used.^33^ Gas exchange values were time-averaged in 30-second intervals, where the highest intervals were considered VO_2_peak.

### Physical strain

Physical strain during walking was defined as the oxygen consumption required when walking at a SSWS, expressed as the percentage of the individual’s maximal aerobic capacity (%VO₂peak). Physical strain was calculated as the ratio of VO₂walk to VO₂peak, multiplied by 100 (VO₂walk/VO₂peak × 100).^10^ Higher physical strain values indicate a greater relative intensity.

### Clinical tests and questionnaire

The 6MWT^15^ was conducted in a 30-m corridor, wearing their usual shoes and using walking aids if necessary. They walked as fast and as far as possible for 6 minutes without running, and the distance covered was measured.^34^ The 6MWT shows high reliability in adults with CP, with a clinically significant difference of 40 m.^35^

The TUG test assesses functional mobility by measuring the time used to stand up from a chair, walk 3 m, turn around, walk back, and sit back down again.^18^^,^^36^ The TUG test is reliable for persons with CP.^37^

The EQ-5D-5L visual analog scale was used to assess QoL. The visual analog scale, where the participants rate their overall QoL on a scale from 0 to 100, where 0 is the worst possible and 100 is the best possible.^38^^,^^39^

### Statistical analyses

All variables were assessed for normal distribution using the Shapiro-Wilk test. Descriptive statistics are presented with means and SD for normally distributed variables, and with medians and interquartile range for nonnormally distributed variables. For descriptive purposes, differences across GMFCS levels were explored through 1-way analysis of variance for normally distributed variables and through the Kruskal-Wallis test for the nonnormally distributed variables. Bonferroni-adjusted post hoc comparisons were applied to account for multiple testing.

The O_2_ cost (mL/kg/m) at preferred speed, and 20% slower and faster walking speeds was evaluated using the Kruskal-Wallis test, for participants (n=83) completing all walking speeds.

Known-groups validity was assessed by comparing physical strain across GMFCS levels using the Kruskal-Wallis test. Post hoc pairwise comparisons with Bonferroni adjustment were conducted to identify differences between GMFCS levels. Independent samples t-test was used to evaluate the difference in physical strain between unilateral and bilateral CP. In addition, the ability of physical strain to discriminate between unilateral and bilateral CP was examined using receiver operating characteristic (ROC) curve analysis with physical strain as the predictor and CP subtype as the outcome. Discriminative performance was quantified by the area under the curve, and interpreted as poor (<0.5), acceptable (0.7-0.8), excellent (0.8-0.9), and outstanding (>0.9), following Hosmer and Lemeshow.^40^

For convergent and discriminant validity, the associations between physical strain and the 6MWT, the TUG test, and QoL were examined. As the residuals from the regression models between physical strain and the 6MWT and TUG were normally distributed, bivariate linear regression was performed with physical strain as the dependent variable. The residuals of the relationship between physical strain and QoL were not normally distributed; however, linear regression was retained to allow comparison across outcomes. The magnitude of the associations (*r*) was considered little (0-0.25), low/weak (0.25-0.49), moderate (0.50-0.69), high (0.70-0.89), and very high (0.90-1.0) following Munro.^41^

Statistical tests were 2-sided, and a significance level of 5% was used. Unadjusted *P* values will be presented, and *P* values adjusted for multiple testing (Bonferroni), *P* value × 3 (number of tests), will be marked with ^ if significant. Statistical analyses were conducted using IBM SPSS Statistics, version 30.0.0.0 (172) (SPSS Inc.).^e^

## Results

### Participants

Four participants did not complete the maximal VO_2_peak test because of high blood pressure, and 2 did not complete the 6MWT because of pain and early discharge from the hospital. A group of 94 ambulant adults (41 men) with spastic unilateral (n=59) or bilateral (n=35) CP, and no intellectual disability, were included. Demographic descriptions of all participants, grouped by GMFCS level, are presented in table 1.

### Walking tests

All participants included in the analysis (n=94) reached maximum effort during the maximal exercise test. The mean VO_2_peak for the entire group was 36.2 mL/kg/min, which reflects a mean of 95% of the age and sex predicted values. VO_2_peak decreased across GMFCS levels, and the difference between GMFCS levels I and III was statistically significant (*P*<.001). The mean VO_2_walk was 17.6 mL/kg/min, and not statistically different between the GMFCS levels (*P*=.093). The median physical strain at SSWS was 50% (18) for the whole group, with higher physical strain for GMFCS levels II (median 56 [13]) and III (median 78 [16]) compared with level I (median 45 [12]). Further, mean physical strain was significantly (*P*<.001) higher for adults with bilateral CP 58(14) than unilateral CP 47(10). See all mean/median values for treadmill and clinical tests, and results across GMFCS levels presented in table 2.

**Table 2:** Results of the maximal exercise test, self-selected walking speed test, functional tests (6MWT and TUG), and quality of life (VAS scale).

Outcome	All (n=94)	GMFCS I (n=64)	GMFCS II (n=22)	GMFCS III (n=8)	Test Statistics	*P* Value	Post Hoc (*P*≤.05)
Maximal exercise test							
VO_2_peak (mL/kg/min)	36.2 (8.6)	38.2 (8.3)	33.7 (8.0)	26.8 (5.5)	8.447	<.001	I-III
Maximum RER	1.08 (0.08)	1.10 (0.07)	1.04 (0.09)	1.03 (0.06)	7.305	.001	I-II, I-III
Maximum HR (beats/min)	182 (12)	183 (11)	182 (14)	174 (14)	1.791	.173	I-III
Maximum lactate (mmol/L)	9.5 (3.0)	10.1 (3.2)	8.4 (2.5)	7.9 (1.9)	4.111	.020	
Predicted VO_2_peak (%)	95 (20)	100 (20)	89 (18)	75 (19)	7.346	.001	
Self-selected walking speed test							
VO_2_walk (mL/kg/min)	17.6 (3.3)	17.1 (2.7)	17.9 (4.3)	19.7 (4.7)	2.435	.093	I-II, I-III
Walking speed (m/s)*	1.12 (0.34)	1.23 (0.19)	0.91 (0.29)	0.51 (0.39)	47.57	<.001	I-II, I-III, II-III
Oxygen cost (mL/kg/m)*	0.25 (0.09)	0.23 (0.05)	0.33 (0.12)	0.65 (0.34)	46.59	<.001	I-II, I-III
Physical strain (%)*	50 (18)	45 (12)	56 (13)	78 (16)	26.51	<.001	I-III, II-III
RER, (†GMFCS III(1))	0.82 (0.05)	0.82(0.05)	0.81 (0.05)	0.89 (0.04)	7.123	.001	I-III
HR (beats/min)*	118 (26)	110 (21)	122 (28)	138 (22)	19.469	<.001	I-II, I-III, II-III
% HRmax (%)*	64 (14)	60 (11)	68 (14)	84 (8)	23.594	<.001	I-II, I-III, II-III
Lactate (mmol/L)*, (†GMFCS I (4), II (1))	1.1 (0.6)	0.9 (0.5)	1.3 (0.7)	3.2 (2)	24.021	<.001	
6MWT (m)*	553 (144)	600 (99)	477 (107)	278 (180)	44.426	<.001	I-II, I-III
TUG (s)*	7.0 (2.3)	6.3 (1.2)	8.0 (2.0)	13.1 (6.0)	38.787	<.001	I-II, I-III
QoL (VAS 0-100), median (IQR), (†GMFCS I(1))	70 (23)	70 (25)	65 (20)	63 (56)	3.046	.218	

NOTE. *P*<.05. Parametric data presented with mean (SD) values, and data tested with 1-way analysis of variance.

Abbreviations: GMFCS, Gross Motor Function Classification System; HR, heart rate; IQR, interquartile range; RER, respiratory exchange ratio.

⁎ Indicates nonparametric data, presented with median (IQR), and data tested with an independent samples Kruskal-Wallis test. Post hoc test was Bonferroni for all tests, GMFCS levels I-III.

† (Number) indicates missing at the GMFCS level mentioned.

The SSWS of the current sample was (mean [SD]) 1.12 m/s (0.34), with slower walking speed for GMFCS levels II and III compared with level I (*P*<.001^). SSWS was confirmed, as the median O_2_ cost of walking was significantly higher for slow (0.26 mL/kg/m [0.07], *P*=.014^) and non-significantly higher for faster (0.25 mL/kg/m [0.06], *P*=.809) walking speed than SSWS (0.24 mL/kg/m [0.08]).

### Known-groups validity

**Fig 2:**
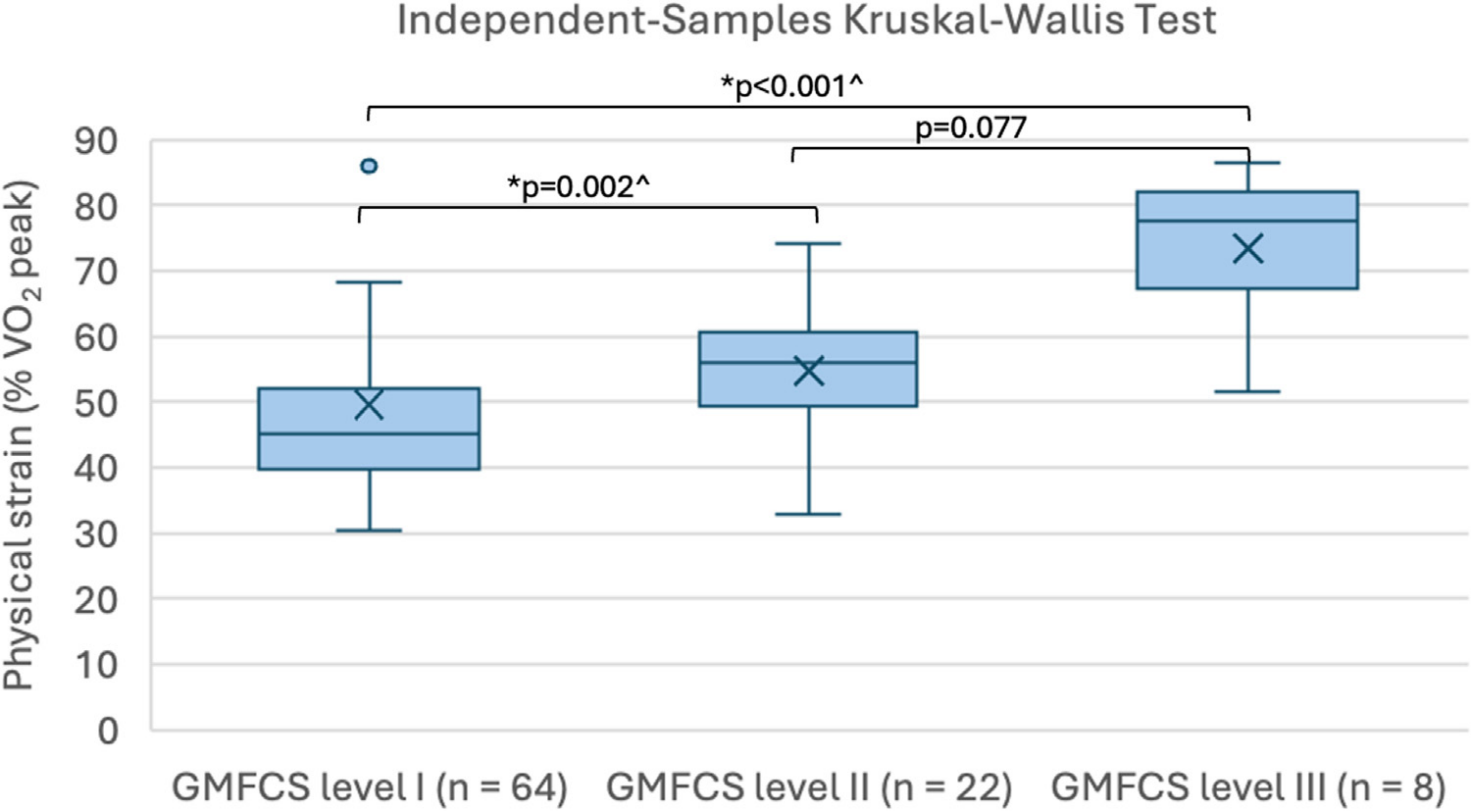
Independent samples Kruskal-Wallis test, with median and interquartile range of physical strain measurements presented for the different GMFCS levels. The whiskers represent the minimum and maximum values within the 1.5 times interquartile range. Values outside this range are represented by dots, indicating outlying values. *P<.05, ^significant when adjusted for multiple tests (Bonferroni). Abbreviations: GMFCS, Gross Motor Function Classification System.

The Kruskal-Wallis test showed significant differences in physical strain for GMFCS levels I and II (*P*=.002^) and between GMFCS levels I and III (*P*<.001^). There were no significant differences in physical strain for GMFCS levels II and III when adjusted for multiple tests (*P*=.077) (see fig 2).

**Fig 3:**
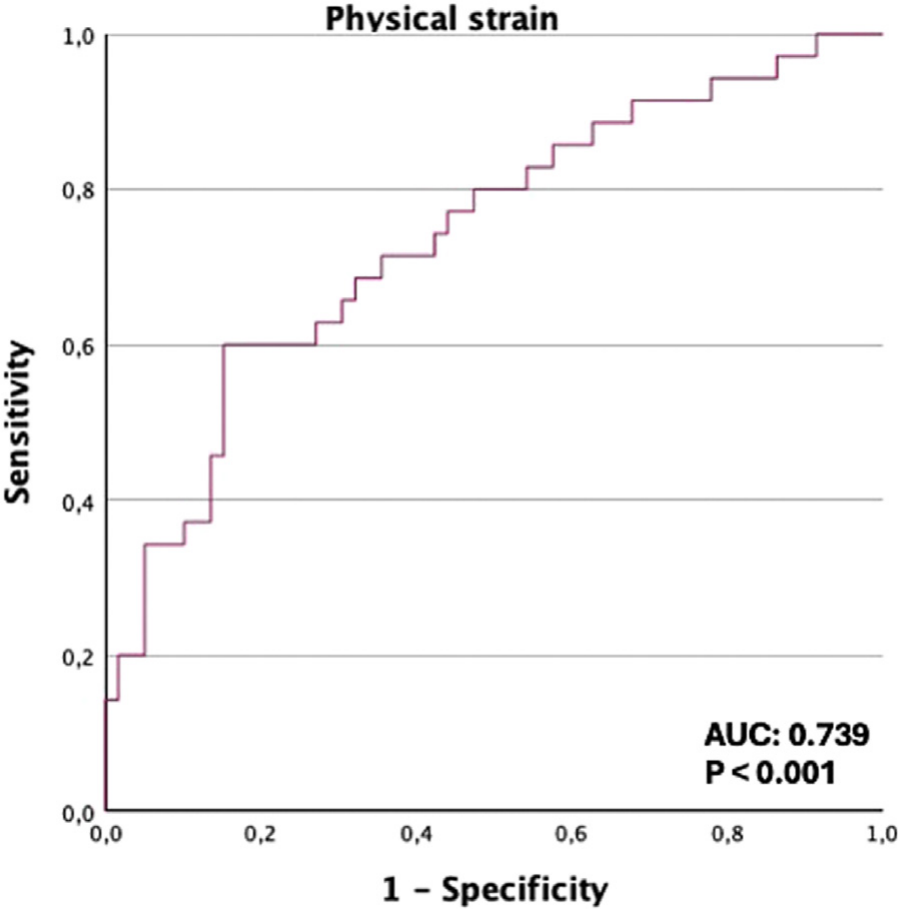
The ability of the physical strain measurement to distinguish between unilateral and bilateral CP groups. The physical strain measurements showed acceptable discriminatory ability. Abbreviation: AUC, area under the curve.

Further, physical strain demonstrated acceptable discrimination between unilateral and bilateral CP subgroups (area under the curve=0.739; SE=0.054; *P*<.001), indicating that individuals with bilateral CP tended to exhibit higher physical strain than those with unilateral CP (see fig 3).

### Convergent and discriminant validity

**Fig 4:**
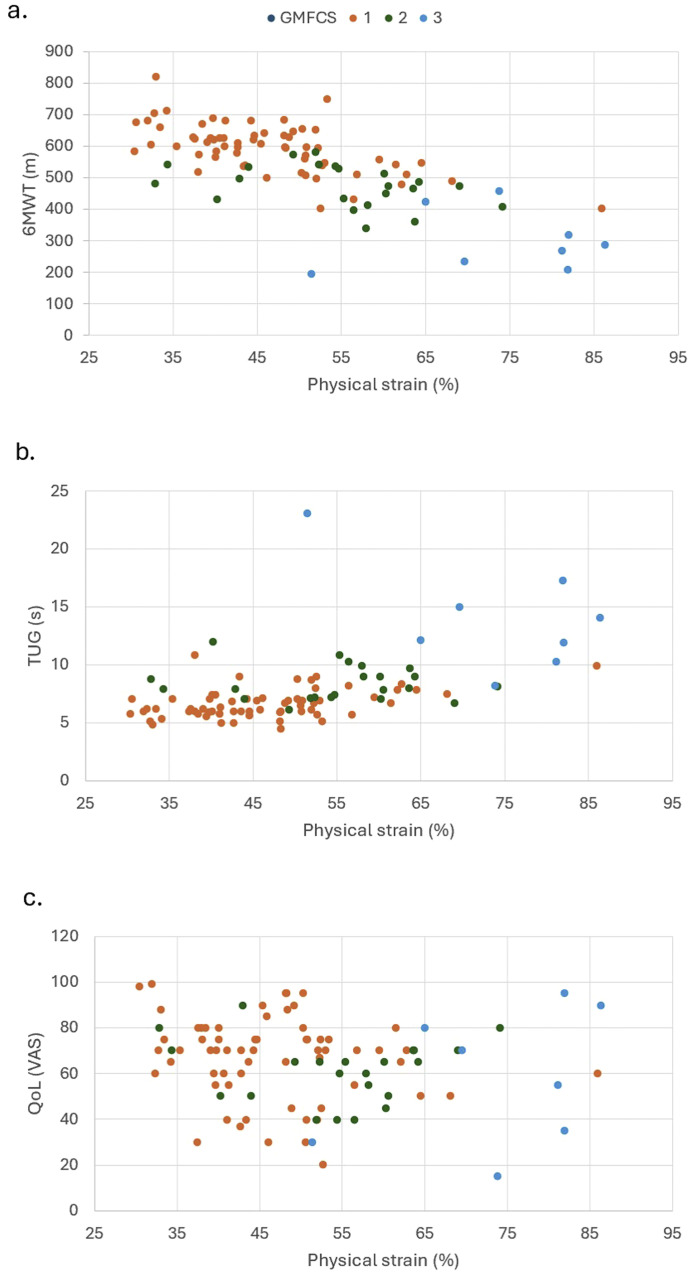
Scatter plots with physical strain (%VO_2_peak) on the x-axis and (a) 6MWT distance (m), (b) TUG time (s), and (c) QoL VAS score (0-100) on the y-axis. Dots are colored by GMFCS level. Abbreviation: VAS: visual analog scale; GMFCS, Gross Motor Function Classification System.

The association between physical strain and 6MWT was strong (*r*=−0.716; *P*<.001). The relationship with TUG was weak-to-moderate (*r*=0.490; *P*<.001), and the relationship with QoL was weak and nonsignificant (*r*=0.157; *P*=.129) (fig 4a-c).

## Discussions

The main findings of this study show increasing physical strain for each GMFCS level for adults with spastic CP, with significantly different physical strain between CP subgroups. As hypothesized, physical strain was strongly inversely associated with functional walking capacity, weakly associated with functional mobility, and not associated with QoL.

### Physical strain of walking

The physical strain is higher (median 50% [18]) in our sample of adults with spastic CP compared with what is previously reported for those without CP (mean [SD] 36% [7.6] with age 59.4 [6.3]),^42^ but comparable with an earlier study for a younger group of adults with bilateral CP.^13^ Physical strain is dependent on both VO_2_peak and oxygen cost of walking in children with CP.^10^ In this study, the overall VO_2_peak is 95% of the age and sex predicted values, but GMFCS level III has significantly lower VO_2_peak compared with GMFCS level I. Furthermore, the VO_2_walk is not significantly different between the GMFCS levels. However, the oxygen cost of walking per meter, which is dependent on walking speed, is significantly different between all GMFCS levels. This indicates that both reduced VO_2_peak and increased oxygen cost of walking contribute to the increasing physical strain of walking in adults with spastic CP with reduced gross motor function.

As physical strain usually increases with increasing walking speed,^43^ SSWS is used to compare between groups and is relevant for walking in daily life. The measured SSWS of the current sample was slower (1.12 m/s) than what is reported for those without CP of 20-69 years (grand mean: 1.35 m/s).^44^ Furthermore, GMFCS level I participants have SSWS and VO_2_peak close to the reference values; however, their physical strain was still elevated, indicating that gait deviations and the resulting higher cost of walking may be responsible.

### Known-groups validity

Physical strain measurements were significantly higher for GMFCS levels II and III compared with GMFCS level I, consistent with previous reports in children with CP^10^ and the subgroups of GMFCS levels I and II in adults with CP.^13^ However, some discrepancies were observed. The higher physical strain in GMFCS level III in this study, compared with that reported by Slaman et al,^13^ may be because of the inclusion of a broader age range, with the highest median age for this group in the current study. Maximum aerobic capacity normally decreases with age,^33^ and this could therefore have influenced the recorded higher physical strain in this group.

Further, 15 participants (8 GMFCS level III, 5 level II, and 2 level I) reported using walking aids occasionally, and 13 reported using a wheelchair (6 GMFCS III, 5 level II, and 2 level I) in some situations. This might reflect that some participants at GMFCS level II were closer to GMFCS level III, which could have influenced the finding of a nonsignificant difference between GMFCS levels II and III. Additionally, handrail support was used by 7 out of 8 persons with GMFCS level III and only by 2 (of 22) persons with GMFCS level II. As handrail support reduces oxygen consumption during walking,^45^ this may have reduced the difference in physical strain between GMFCS levels II and III.

Physical strain measurements also demonstrated acceptable discriminatory ability between unilateral and bilateral spastic CP subgroups. Individuals with bilateral CP exhibited higher physical strain compared with those with unilateral CP. These results align with relatable results of studies of adults with CP, indicating more pronounced gait deviations in bilateral CP, leading to a shift in propulsion strategy.^46^

### Convergent validity

The strong inverse association between physical strain and 6MWT distance indicates that both measures capture the same underlying construct, functional walking capacity, where both measures reflect how efficiently an individual can sustain walking relative to their available aerobic resources. Although the 6MWT was originally designed to assess functional capacity,^15^ VO_2_peak alone is not reported to predict 6MWT distance in this population.^16^ In this sample, VO_2_peak was relatively high, suggesting that elevated oxygen consumption during preferred walking, rather than low aerobic capacity, is a key contributor to increased physical strain. Known predictors of 6MWT distance, such as reduced plantar flexor strength,^47^ hamstrings tightness,^17^ and the association with gait deviations^48^ may also contribute to higher physical strain. Differences in VO_2_peak between GMFCS levels, however, likely further explain the higher strain in levels II and III. Together, these findings support the convergent validity of physical strain as a measure of functional walking capacity.

### Discriminant validity

The relationship between physical strain and the TUG test was weak-to-moderate, indicating that these measures represent different constructs. Whereas physical strain and 6MWT both reflect functional walking capacity, the TUG test reflects a broader mobility component,^37^ including more advanced functional abilities (muscle strength, balance, and turning).^37^ Walking on a tilted treadmill may still challenge some participants,^49^ but it requires less of the complex functional abilities involved in the TUG test. Furthermore, the TUG test relies less on sustained aerobic demand. This explains the weak-to-moderate association and supports the discriminant validity of the physical strain measure.

Despite prior findings linking reduced physical function to decreased QoL in adults with CP,^46^^,^^50^ this study found no relationship between physical strain and QoL. Furthermore, the lack of relationship reflects that multiple factors beyond the physical strain of walking influence QoL in this population; comorbidities,^51^ pain,^52^^,^^53^ and fatigue,^54^ among others. This aligns with the study’s hypothesis, that QoL reflects broader physical, psychological, and social dimensions^55^ not captured by physical strain measurements.

### Study limitations

This study examined the associations between physical strain and functional or clinical measures, and did not adjust for possible confounders, which is a limitation of the approach. Nevertheless, because the objective was to assess the construct validity of physical strain through hypothesis testing, this methodology was considered appropriate.

This study included an uneven distribution of participants across GMFCS levels and CP subgroups, making the findings more generalizable to individuals with higher gross motor function. The small number of participants in GMFCS level III likely contributes to the large variability and the nonsignificant difference between levels II and III. In addition, although participants in GMFCS level III typically use walking aids in daily life, they did not during treadmill testing; however, most used handrail support, making the test conditions more comparable to walking with aids in daily life.

The combination of increased physical strain, reduced SSWS, and adequate maximum aerobic capacity suggests that biomechanical constraints may increase oxygen consumption during walking in this group. Future studies should identify which biomechanical constraints affect oxygen consumption at SSWS, and evaluate the reliability and responsiveness of the physical strain measurements.

## Conclusions

Adults with spastic CP exhibited higher physical strain with increasing gross motor function impairments. Physical strain differed between unilateral and bilateral spastic CP, supporting the known-groups validity. As hypothesized, physical strain showed a strong inverse association with functional walking capacity (6MWT), a weaker association with mobility (TUG), and no association with QoL. Overall, these findings support the construct validity of physical strain as an indicator of functional walking capacity in adults with spastic CP.

## Suppliers

a. PPS med series; Woodway.
b. Tango M2; SunTech Medical.
c. Quark CPET; COSMED.
d. Biosen C-Line; EKF-diagnostic.
e. SPSS, version 30; IBM.

## Disclosure

The investigators have no financial or nonfinancial disclosures to make in relation to this project.
